# Dual-branch feature encoding framework for infrared images super-resolution reconstruction

**DOI:** 10.1038/s41598-024-60238-9

**Published:** 2024-04-23

**Authors:** Yuke Zhang, Peizi Zhou, Lizhu Chen

**Affiliations:** Tropical Agriculture and Forestry School, Hainan University, Haikou, 570228 China

**Keywords:** Electrical and electronic engineering, Computer science, Information technology, Software

## Abstract

Infrared thermal imaging is a passive non-contact detection and identification technology, which is not subject to electromagnetic infection and good concealment, is widely used in military and commercial fields. However, due to the limitations of the existing infrared imaging system mechanisms, the spatial resolution of the acquired infrared images is low and the edge details are blurred, which in turn leads to poor performance in downstream missions based on infrared images. In this paper, in order to better solve the above problems, we propose a new super-resolution reconstruction framework for infrared images, called DBFE, which extracts and retains abundant structure and textual information for robust infrared image high-resolution reconstruction with a novel structure-textual encoder module. Extensive experiment demonstrates that our proposed method achieves significantly superior contraband high-resolution reconstruction results on the multiple dataset compared to progressive methods for high resolution infrared image reconstruction, effectively proving the practicability of the method proposed in this paper.

## Introduction

Infrared thermal imaging uses signal processing, photoelectric conversion and other technical measures to convert infrared radiation in the target area into visual images^1^. It has the advantages of round-the-clock operation, good anti-jamming and not easy to be detected^2^, therefore, it is widely used in medical images^3^, industrial equipment^4^, security equipment^5^ and military operations^6^.

However, due to the weak thermal radiation of the imaging target itself and the physical limitations of the infrared detectors themselves^7^, the image quality of infrared images output from typical infrared sensors is low. The resolution of low-quality infrared images is usually lower than that of visible light images, and is often accompanied by problems such as susceptibility to noise carryover, blurred and missing edge details, which will greatly affect downstream tasks based on infrared images. For example, in IR graphics-based target detection tasks, these drawbacks greatly diminish the saliency of target features in the image and are not conducive to detecting targets with complex backgrounds^6^.

In order to improve the image quality of infrared thermography, we can directly use infrared detectors with high focal plane detection performance. However, high-performance infrared(IR) detectors are expensive and are not conducive to wide application. Therefore, researchers have turned to the use of software algorithms to improve the spatial resolution of infrared images, which is also known as the image super-resolution reconstruction (SRR) technique^8–10^. This method is a more viable option due to its low cost, good generalisation and direct application to the original imaging device without upgrading the hardware of the infrared thermal imaging device. The application of image SRR techniques allows existing IR imaging systems to capture clearer, more detailed, high-quality images, greatly improving the ability to analyse and interpret IR images for more accurate and informed decision-making.

In the early stage, researchers mostly used reconstruction-based methods^11^ to solve the problem of super-resolution reconstruction of infrared images.For instance, Hui^12^ et al. proposed a single infrared image SRR algorithm that combines non-local means (NLM) and steering kernel regression (SKR). In this algorithm NLM denoises each pixel in the image by finding similar pixels in its neighbourhood and performing weighted averaging (the weights are computed based on the similarity between pixel neighbourhoods).SKR assigns weights to each pixel by calculating the local similarity and consistency of geometrical and radiometric structures between pixels in the neighbourhood. These weights are used to compute the local regression of the pixels, which effectively suppresses noise and preserves the edges of the infrared image. However, the iteration of this algorithm requires constant updating of the NLM and SKR weight matrices, and its computational complexity is high. Meanwhile, the performance of the method may depend on the tuning of parameters, such as the global filter parameters of NLM, the smoothing parameters of SKR, the step size and the regularisation parameters, which leads to the low robustness and generalisation ability of the algorithm. Zhao et al.^13^ proposed a new method for super-resolution infrared image reconstruction based on sparse representation from the data perspective. The method assumes that a pair of dictionaries can be obtained such that the low-resolution (LR) image block shares the same sparse representation with the high-resolution (HR) image block, so that the HR image block can be reconstructed from the sparse representation of the LR image block. Where stable dictionary pairs are obtained by classifying the training samples into multiple clusters and training two dictionaries on these clusters. This method’s requires two trainings to obtain stable dictionary pairs, but the sparse decomposition is unstable, which in turn leads to artefacts in the reconstruction of image blocks for structures with complex textures. In order to solve the instability problem of the sparse decomposition of the above methods, Mao et al.^14^ proposed a super-resolution reconstruction method for infrared images based on Compressive Sensing (CS) theory. It integrally adopts the image degradation model, the sparse transform method based on differential operation and the Orthogonal Matching Pursuit algorithm to transform the image super-resolution reconstruction problem into the sparse signal reconstruction problem in CS theory. The biggest advantage of this algorithm is that the sparse transform of HR image is obtained directly through the spatial distribution properties of the image, instead of training redundant dictionaries to accelerate the computation speed, which eliminates the dependence on samples and can obtain good reconstruction results quickly.

However, these reconstruction-based techniques have limited feature extraction capability and do not maximise the reconstruction using the information contained in the LR infrared images, and therefore have limited accuracy in reconstructing the details of infrared images. Fortunately, with the recent advances in deep learning and CNN theory, more and more super-resolution networks designed specifically for infrared images have been proposed because of their powerful feature extraction capabilities^15–25^.

For instance, Zou et al.^26^ a method for super-resolution reconstruction of infrared images using a convolutional neural network with skip connections. The network structure contains several convolutional and anti-convolutional layers for extracting and recovering features of infrared images, respectively. Skip connections and channel fusion are introduced into the network to increase the number of feature maps and to facilitate the recovery of image details by the deconvolutional layers. The method attempts to increase the number of feature maps by introducing global residual learning and local residual learning, which in turn solves the problem of insufficient dependence of features on different scales of information context.He et al.^17^, on the other hand, wanted to start from the perspective of features at different scales and proposed a cascade architecture of two successive deep networks. In this framework, an intermediate point is set to divide the input LR image features into two parts: large-scale structural features and small-scale detail features, and the large-scale structural information is reconstructed by the first network and the small-scale image details are reconstructed by the second network. These two networks encode features at different scales and are jointly trained by a multi-scale loss function. In this way, infrared images can be reconstructed more accurately even with simpler deep networks. However, the two networks of this method only use a fixed one receptive field for feature extraction, so it may lead to the problem that the networks have insufficient information at different scales since the global and local features. In detail, while expanding the receptive field for extraction of large-scale information, the minute details of the large-scale information are ignored, so they may be smoothed over a large range of contexts, which in turn leads to a blurring of the structural theory of the objects in the image. The opposite is true for small-scale information.

The two latest works also continue the same idea as the above two articles, hoping to enhance the super-resolution reconstruction effect of infrared images from the global nature of features and the expression level of features. Specifically, Yuan et al.^27^ proposed a Gradient Residual Attention Network (GRAN), which uses a Residual Dense Block to extract deep features, a Gradient Operation to obtain fine-grained detailed features, and a 3D Attention Block to learn the channel and spatial correlation of features. Block to learn the channel and spatial correlation of features to selectively capture more useful information features. The model makes full use of the depth features and fine-grained features in the LR image, which effectively improves the quality of super-resolution reconstruction of IR images.The GRAN model contains multiple dense convolutional layers and attention modules, which may lead to high computational cost, especially when processing large images.Qin et al.^23^ argued that the pixel distribution of the IR image is uniform and the gradient range is limited, which poses a challenge for the model to capture the effective feature information poses a challenge. They therefore constructed a new super-resolution reconstruction model for infrared images based on the Transformer model, called LKFormer.The model aims to explicitly model local and global range dependencies in infrared images, for which a Large Kernel Residual Depth Convolutional Attention (LKRDA) module with linear complexity is designed, which mainly uses deep convolution of a large kernel for non local feature modelling as an alternative to the standard self-attention layer.

When we review the above methods, we find that the core idea of these methods is to try to make full and effective use of the information contained in the existing low-resolution infrared images to complete the reconstruction. However, they either focus on the high-frequency information but neglect the influence of low-frequency information on super-resolution reconstruction, which precisely determines the clarity of the edge contour and texture structure of the objects in infrared thermal images. Or, when focusing on both high-frequency and low-frequency information, they ignore the fact that these information should also be encoded in combination with local or global features, which results in the loss of small structural details or inconsistency of global texture information.

To address the above problems, we try to explore a more reasonable reconstruction network to solve the challenges in feature extraction and reconstruction. We hope that the HR infrared image reconstruction model can pay more attention to the balance of the feature extraction module, balancing the parameter weights of low-frequency features and high-frequency features, thus avoiding the loss of low-frequency information in the forward transfer of the network. We also hope to pay more attention to the contextual association of high-frequency and low-frequency features to avoid unnecessary information loss.Therefore, we designed a new dual branching feature encoding (DBFE) framework which encodes high and low frequency features separately. The purpose of separate coding is to reduce the correlation between low and high frequency features in order to facilitate more adequate extraction of texture and geometric structural features from each frequency information. Second, in order to better adapt to the two-branch structure, extract more global information for image reconstruction, and avoid local bias, we design a new Structure-Textual Encoder (STE) module to establish the local dependence and remote dependence of the feature information, which expands the sensory field while enhancing the correlation of the extra-local feature signatures information.The STE enables us to to perform accurate image reconstruction using rich structural and contextual features. Structural and contextual features for accurate high-resolution reconstruction of infrared images. Meanwhile, the dual-branch feature coding framework proposed in the paper greatly reduces the difficulty of texture feature and geometric structure feature extraction, and solves the feature loss problem of traditional reconstruction networks in the forward propagation process.

To this end, we propose a dual-branch feature coding (DBFE) framework, in which global texture feature and geometric structure feature extraction is achieved by STE and high-resolution infrared images are reconstructed by a decoder. Our contributions can be summarised in two aspects:

- For the shortcomings of infrared images, such as missing texture and geometric structure features and low contrast, we propose a new dual-branch feature coding framework for single infrared image super-resolution reconstruction method
- Aiming at the problem of poor extraction and representation of global diversity features, we design a plug-and-play STE. the STE can establish the local and remote dependencies of feature information in parallel, which effectively avoids the problem of the loss of global texture features and local geometric structure features. The results of ablation experiments show that the STE helps to enhance the expression of texture features and geometric structure features of the model.

## Related work

In recent years, deep learning-based methods have achieved significant advantages over traditional methods in the problem of visual image super-resolution reconstruction. On this basis, researchers have also relied on visible light resolution-producing reconstruction techniques to solve the problem of super-resolution reconstruction of infrared images. Therefore, these two types of algorithms are closely related to each other and their research progress is also closely related. That is why we will introduce these two types of algorithms separately in this subsection, discussing their respective characteristics and inspirations for our proposed algorithm.

### Deep networks for visible image super-resolution

The utilisation of deep learning based SRR methods is gaining attention in the field of computer vision. These techniques have proved to be very effective in improving the resolution and quality of images and in improving visual perception and analysis.Dong et al.^23^ proposed the first deep learning based image super resolution algorithm SRCNN in 2014.This method first uses an interpolation algorithm for up-sampling and then a deep neural network is used to refine the image but the pre-up-sampling introduces artefacts and noise amplification. Shi et al [54] proposed the ESPCN model. This model directly uses sub-pixel convolution at the end for up-sampling, which enables the low-resolution space to retain more texture information, and this up-sampling method can obtain better reconstruction results. However, the model only studies the problem of up-sampling and does not address the problem of how to learn to utilise the rich feature texture information.

To further improve the visual quality of reconstructed images, Generative Adversarial Networks (GAN)^28^ were introduced into the SRR domain. Lediger et al [56] proposed the SRGAN model which uses generative adversarial networks for SRR tasks.SRGAN designs SRResNet as a generative network with 16 ResNet^29^ blocks. Wang et al.^30^ proposed ESRGAN model which extracts features by removing batch normalisation and adding dense structure. With the great success of the Transformer framework in natural language processing, it has also been introduced to the task of image superscoring variability reconstruction and has made great progress. swinIR model proposed by Liang et al.^31^ is the first superscoring model based on the Transformer framework, which combines Transformer with CNN in the feature extraction phase in the feature extraction stage. Thanks to Transformer’s ability to construct extremely strong remote dependencies and CNN’s ability to extract local features, SwinIR obtains excellent hypersegmentation reconstruction results.

### Deep networks for infrared image super-resolution

With the success of deep learning basedvisible image SRR methods on visible images prompted many researchers to use them for infrared images. Inspired by the algorithm used for visible image SRR, the, He et al.^17^ in 2019 proposed a deep neural network cascade architecture with multiple receptive fields to improve the spatial resolution of infrared images to a large scale factor. In order to make full use of the information of neighbouring points, preserve the image structure and avoid step artifacts, Liu et al.^18^ proposed a super-resolution reconstruction method for infrared images based on quaternion total variation and high-order overlapping group sparse, but the floating-point operation of this algorithm is too complicated. Zou et al.^26^ proposed SRR of infrared images based on a convolutional neural network with skip connections, which extracts image features and recovers image details through convolutional and inverse convolutional layers. Yang et al.^32^ proposed Spatial Attention Residual Network (SAResNet), which consists of spatial attention and residual blocks to achieve high accuracy infrared image SRR.

Yao et al.^33^ proposed a new super-resolution method for infrared images based on discriminative dictionaries and deep residual networks, which benefits from the advantages of compressed sensing and deep learning to obtain more accurate reconstruction results. Zou et al.^34^ proposed a super-resolution imaging algorithm for infrared images based on auxiliary convolutional neural networks, which utilises the detail information provided by visible light images in low light conditions for super-resolution imaging of infrared images.

With the RGB-IR cross-input and sub-pixel upsampling networks being proposed, Du et al.^19^ improved the spatial resolution of infrared (IR) images by combining them with colour images with higher spatial resolution obtained with different imaging modalities. Due to the limitations of IR imaging principles and imaging systems, collected IR images usually encounter many problems, such as low resolution, insufficient detail information and blurred edges. Liu et al.^21^ proposed a super-resolution reconstruction method for infrared images based on generative adversarial network. But the reconstruction network based on GAN model intends to introduce artefacts in the infrared images. Liu et al.^21^ proposed an SRR model for infrared images based on a generative adversarial network with an attentional mechanism, where the generator is designed to recursively access the network while improving the loss function in order to recover the texture details of the infrared images. Furthermore, Prajapati et al.^35^ proposed a channel segmentation based convolutional neural network (ChasNet) for extracting high frequency features from infrared images using channel segmentation.

Throughout the above studies, the core idea of this series of work always revolves around the purpose of enhancing the feature extraction capability of the network. While existing work does not adequately consider the local dependence and global dependence of features at different scales in extracting high-frequency features and low-frequency features. Therefore, we propose a simple but effective Structure-Textual Encoder module, which establishes a feature extraction model with more balanced local dependence and global dependence on features from the perspectives of structural and texture feature extraction and contextual dependence.

## Method

### Motivation

We found that the blurring of colour-monotone infrared images is mainly caused by unclear geometric structures and missing texture features in the images. Meanwhile, we investigated the existing IR super-resolution reconstruction networks, which all try to enhance the clarity of geometric structures and texture features in the reconstructed images by enhancing the extraction capability of high and low-frequency features or by enhancing the contextual dependency between features.

**Figure 1:**
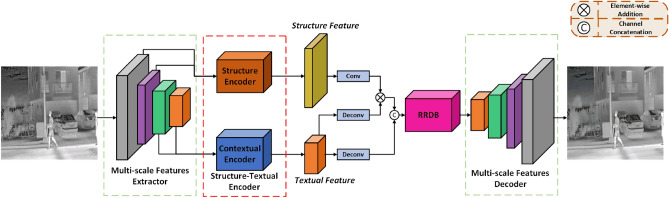
The framework of the proposed DBFE. We employ the pre-trained VGG19 as a muti-scale features extractor to acquire features at different levels. Structure-contextual encoder is a plug-and-play module that we propose mainly for obtaining structure and contextual features information at different hierarchical multi-levels.

However, they do not simultaneously consider enhancing the extraction capability of high and low frequency features and the contextual dependency of features. As a result, existing algorithms are often insufficient to adequately reconstruct texture and geometric structure features in infrared images.

This inspired us to reconstruct high-resolution infrared images using separate encoding of geometric structure information from shallow feature layers and texture information from deep feature layers. To this end, we design a Dual Branching Feature Encoder framework (DBFE) for encoding geometric structure features and texture features from different layers separately to avoid the loss of different feature information. Further, to avoid local bias in reconstructed images, we design a new Structure-Textual Encoder(STE) module for establishing global attention and remote dependency of feature information, which effectively integrates geometric structural and texture detail features in multi-scale large-scale images to enhance the detail features of reconstructed infrared images in various scenarios.

Our proposed STE module applied to a two-branch encoder framework aims to robustly reconstruct high-resolution infrared images in a variety of scenarios by multiplexing the same multiple features at multiple levels and obtaining features with different levels of geometric structure and texture detail.

### Overview of the DBFE framework

Figure 1 presents the proposed dual-branch feature encoding (DBFE) Framework. The core idea of DBFE is to encode the structural and textural features in the original infrared image separately, as a way to achieve the extraction and retention of different levels of features in the sub-channels.

In DBFE, we first use a pre-trained Multi-scale Feature Extractor for feature extraction. Then, we divide the extracted features into low-level and high-level features and send them to SCE for encoding separately. A well-designed SCE module is capable of deeply extracting otherwise sparse and unobtrusive structural and textural feature information for the purpose of enhanced network feature extraction and fixation.

Finally, we fused the structural and contextual features obtained from SCE coding and fed them to the RRDB module. The RRDB module, after further computation of the obtained fused features, outputs them to the Decoder, which finally reconstructs the data stream into a high-resolution infrared image.

### Structure-contextual encoder

**Figure 2:**
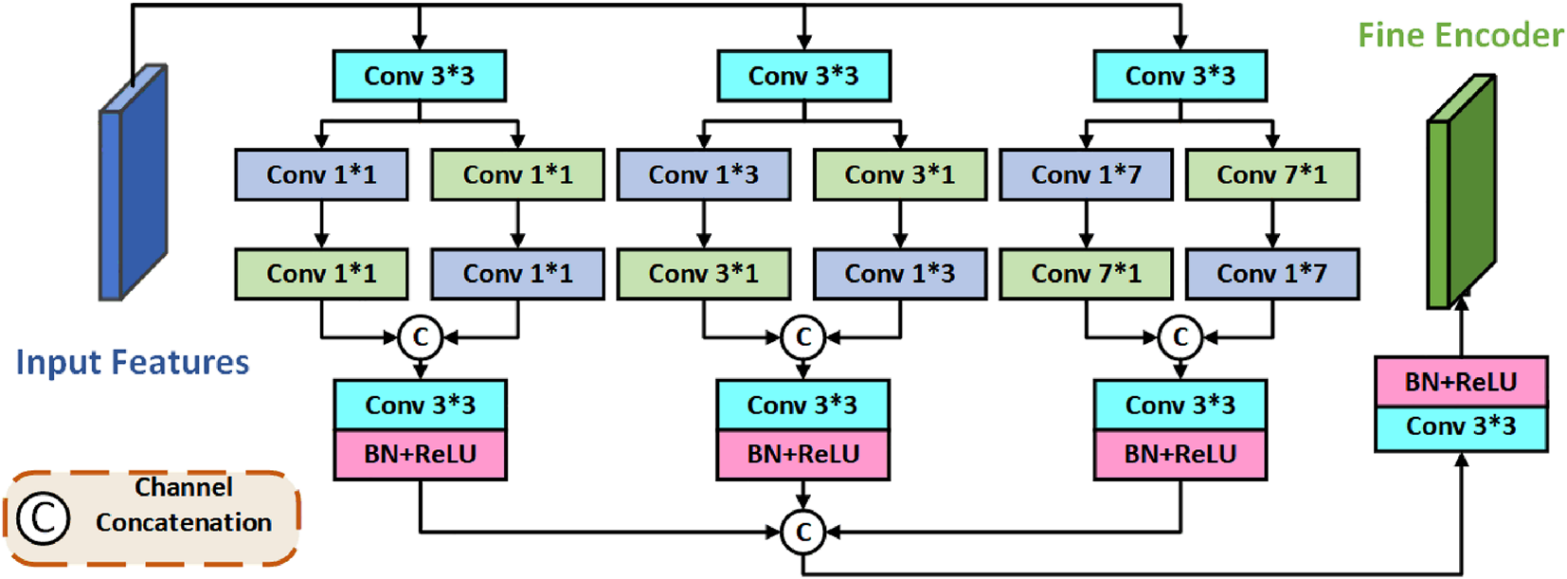
The global feature dependency integration module. The input features are passed through three parallel GFDI blocks and the outputs of all GFDI blocks are fused to have globally relevant features. The internal structure of each GFDI block is similar in that it has a parallel branching structure. Notably, the scale of the convolutional blocks in the internal branching structure of each GFDI block is different. The purpose of setting up convolutional blocks of different sizes is to establish connections between features of different scales to form global correlations.

STE consists of Structure Encoder(SE) and Textual Encoder(TE), SE is used to extract low-level structure features, TE is used to extract high-level contextual features. Both SE and TE use the same Global Feature Dependency Integration(GFDI) module, the detailed structure of which is shown in Fig. 2.

The input features are passed through three parallel GFDI blocks and the outputs of all GFDI blocks are fused to have globally relevant features. The internal structure of each GFDI block is similar in that it has a parallel branching structure. Notably, the scale of the convolutional blocks in the internal branching structure of each GFDI block is different. The purpose of setting up convolutional blocks of different sizes is to establish connections between features of different scales to form global correlations.

The GFDI module aims to efficiently extract rich structure and contextual feature information from different layers of features and global dependencies between them for robust high-resolution infrared image reconstruction.

The GFDI module consists of three GFDI blocks, and the mathematical expression for any one of the GFDI blocks is:

1 $$\begin{aligned} F_{i} = \Re [Conv_{3\times 3}(f_{l}(Conv_{3\times 3}(x)))\oplus (f_{r}(Conv_{3\times 3}(x)))]. \end{aligned}$$

where *x* denotes the input features of the GFDI block and $F_{i}$ is the integration feature output by the *i* GFDI block, where *i* takes the values 1, 2, 3. $Conv_{3*3}$ denote the local convolutions with a $3\times 3$ kernel. $\Re$ represents the batch normalisation and ReLU operations. The symbol $\oplus$ represent the concatenation operation. Where $f_{l}$ and $f_{r}$ represent the convolution process of the two branches, which can be expressed as respectively:

2 $$\begin{aligned} f_{l}= & {} Conv_{m\times 1}(Conv_{1{\times }m} (Conv_{3\times 3}(x))), \end{aligned}$$

3 $$\begin{aligned} f_{r}= & {} Conv_{1{\times }m}(Conv_{m{\times }1} (Conv_{3\times 3}(x))). \end{aligned}$$

where $Conv_{1{\times }m}$ and $Conv_{m{\times }1}$ represent convolution operations where the convolution kernel is $1{\times }m$ and , respectively, and takes the values 1, 3, 7.

After getting the output of each GFDI block, we can integrate to get the overall output of GFDI as:

4 $$\begin{aligned} F= \Re (Conv_{3\times 3}(F_{1}{\oplus }F_{2}{\oplus }F_{3})). \end{aligned}$$

### Loss function

The model proposed in this paper follows the basic paradigm of GAN models. Our proposed DBFE is the generator, while the structure of the discriminator discriminator model used for training is consistent with that in the literature^36^ and will not be discussed here.

In order to make the model stable for training, we combine the perceptual loss^37^, content loss^38^ and adversarial loss^39^ to train the model. Firstly, we use the perceptual loss to construct the high dimensional features, the mathematical expression of which is shown in Eq. (5).

5 $$\begin{aligned} L_{per}={\frac{1}{W{\times }H}}\sum _{W}^{x=1} {\sum _{H}^{y=1} {(\phi _{i}(I_{HR})_{x,y}-\phi _{i}(G(I_{LR})_{x,y}))^{2}}}. \end{aligned}$$

where *H* and *W* are the width and height of the image and are used to describe the dimensions of the image; It is worth noting that in deep networks, using post-activation feature values tends to lead to sparse high-frequency features, producing redundant feature maps that degrade network performance. We use pre-activation feature values to construct the perceptual loss. In deep networks, using post-activation feature values tends to result in sparse high-frequency features, which produces redundant feature maps that degrade network performance. Therefore, we use pre-activation feature values to construct the perceptual loss.

In addition, the perceptual loss is better for image texture reconstruction, but it is prone to cause detail tolerance artefacts in the image, and this defect is amplified in infrared image images. To solve this problem, we add content loss to the loss function:

6 $$\begin{aligned} L_{\delta }(I_{HR},I_{SR})= \left\{ \begin{array}{l} {\frac{1}{W{\times }H}}(I_{HR}-I_{SR})^{2},\ I_{HR}-I_{SR}\le {\delta } \\ {\delta }{\vert }I_{HR}-I_{SR}{\vert }-{\frac{1}{W{\times }H}}{\delta }^{2},\ otherwise \end{array}\right. \end{aligned}$$

Many of the image super-resolution models in the current study use the L2 loss function, which is directly related to the objectively assessed metric Peak Signal-to-Noise Ratio (PSNR), and the L1 loss function, which allows the model to converge more quickly. However, the L1 used for neural network training always has a large gradient, and although it can converge the model stably, it tends to miss the minimum at the end of the process. On the other hand, the L2 loss function penalises outliers more and has a parabolic structure that opens upwards, and it converges more slowly near the minimum. So we combined the advantages of both and constructed the content loss.

Finally, we used the Wasserstein distance to describe the distance between the generated image and the real image. Thus, the gradient constraint is added to the adversarial loss of the original GAN. The adversarial loss is shown in Eq. (7):

7 $$\begin{aligned} L_{adv}=E[D(I_{HR})-D(I_{SR})]+{\eta }E[{\Vert }{\triangledown }_{1} D(T)_{2}-1{\Vert }^{2}]. \end{aligned}$$

where the first term on the right side of the equation describes the distance between the super-resolution image and the original high-resolution image as judged by the discriminator, the second term is the penalty gradient, and $\eta$ is the penalty coefficient.

In summary, the overall loss function of the model proposed in this paper is:

8 $$\begin{aligned} L=L_{adv}+\lambda {L}_{\delta }+{\mu }L_{per}. \end{aligned}$$

where $\lambda$ and $\mu$ denote the balancing parameters for $L_{adv}$, $L_{\delta }$ and $L_{per}$, respectively.

## Experiments

### Experimental data

In this paper, the high-resolution CVC-09/ CVC-14^40^ dataset provided by FIR is used for model training. The image content of this dataset mainly includes streets, pedestrians, cars, traffic indications, and houses and buildings. During training, we degrade the high-resolution infrared images into low-resolution infrared images to form data pairs for supervised training of the model.

In the testing phase, in order to test and evaluate the model performance, we randomly select real IR images from three commonly used datasets, namely MSRS^41^, TNO^42^ and RoadScene^43^, as the test set. This can effectively isolate the training set and the test set to ensure the effectiveness and fairness of the experimental evaluation. In the testing stage, two test sets were set up in order to comprehensively test and evaluate the model performance. The first test set consists of three commonly used real IR image datasets, MSRS^41^, TNO^42^ and RoadScene^43^. The second test set consists of the IR700 dataset^26^. All the test data are not included in the training set, which can effectively isolate the training set and the test set and ensure the validity and fairness of the experimental evaluation.

### Network model parameter setting and evaluation indicators

#### Implement details

The optimisation process uses the Adam optimiser where the decay rate $\beta _{1}=0.9$ and $\beta _{2}=0.95$. Learning rate $lr=0.0001$. We trained the model for 300 epochs and activated linear learning rate decay after 150epochs. The weights were initialised using a normal distribution to generate values that conform to a standard deviation of 0.02. The models covered in this paper were implemented using the PyTorch framework and the models were trained and tested using two GeForce RTX 3090Ti.

#### Metrics

In traditional image super-resolution tasks, performance metrics commonly used to measure system performance include Peak Signal-to-Noise Ratio (PSNR)^44^ and Structure Similarity Index Measure (SSIM)^45^. In this paper, these performance metrics are also used to judge the detection capability of the model.

### Experiments result and analysis

#### Evaluation on the first test set

Since the vast majority of super-resolution reconstruction algorithms for infrared images do not have open-source code, we have only been able to compare the algorithm proposed in this paper with five classical and state-of-the-art general-purpose super-resolution reconstruction algorithms. The specific algorithms used for comparison are Bicubic^46^, EDSR^47^, SRGAN^38^, R-ESRGAN^36^ and SwinIR^31^. In order to make an objective and fair comparison, the algorithm codes we used with the performance comparison were downloaded from Github and set up according to the configuration requirements, and some of the procedures were adjusted without affecting the algorithm performance analysis. Also, the hyperparameters of all algorithms were retrained using the same training dataset.

The main goal of our work is to perform high-resolution reconstruction of infrared images. We tested the performance of the five network models used for comparison on the test sets constructed by MSRS, RoadScene and TNO. Table 1 shows the results of the quantitative analyses, where the objective evaluation metrics PSNR and SSIM values obtained by the DBFE Framework proposed in this paper are higher than those of the other algorithms tested on the three datasets.

**Table 1:** The results of the comparisons with other model.

Dataset	Metrics	Bicubic	EDSR	SRGAN	R-ESRGAN	SwinIR	Ours
RoadScene	PSNR	23.01	26.16	25.03	27.78	26.79	28.97
	SSIM	0.75	0.836	0.77	0.84	0.88	0.90
TNO	PSNR	26.45	29.97	28.07	30.63	29.71	31.23
	SSIM	0.70	0.79	0.75	0.81	0.85	0.88
MSRS	PSNR	22.54	23.83	22.59	24.19	24.32	26.37
	SSIM	0.63	0.67	0.65	0.69	0.70	0.75

This table shows the performance metrics comparing the infrared image super-resolution results of the proposed DBFE in this paper and other infrared image super-resolution reconstruction algorithms.

**Figure 3:**
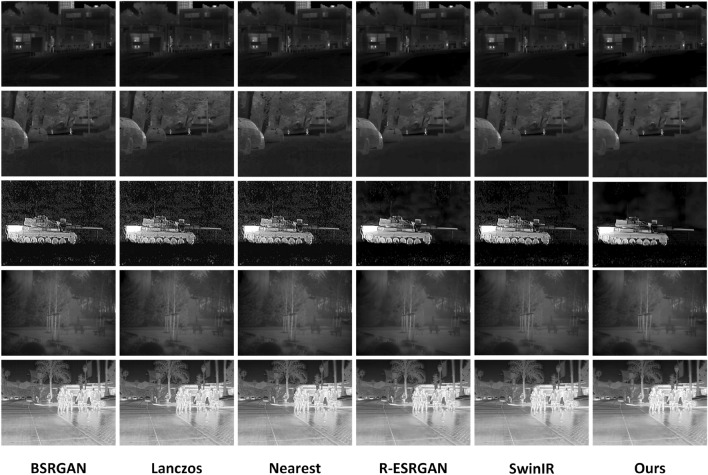
Infrared image super-resolution reconstruction results. The above figure shows the reconstruction results of DBFE proposed in this article. We can clearly see that DBFE performs well in various scenarios and has good reconstruction performance for Infrared image super-resolution.

Figure 3 shows the qualitative comparison of our method with other methods. From the visual analysis of the experimental results, the reconstructed image by the traditional Bic method is overall fuzzy and accompanied by noise, while the reconstruction quality of the other algorithms is better than that of Bicubic and is sufficient to obtain a clear texture for regions with obvious boundaries.

The second, third and fifth columns in Fig. 3 are the reconstruction results of EDSR, SRGAN and SwinIR, respectively, and we found that the reconstruction results of these three algorithms are better than those of Bicubic, but there are still artifacts and a lot of noise in the reconstructed infrared images (which can be found from the observation of the third row of images). At the same time, these three methods do not restore the structural information of the image well, for example, we observe the image in the second row and find that the car contours reconstructed by these three algorithms are blurred and the geometrical relationship is not clear.

Further observation and comparison, the R-ESRGAN model is better than the above three algorithms, and the reconstructed image has no obvious noise and artefacts, but it is too smooth for some homogeneous regions and lacks some details of realism. We can clearly find in the third row and fourth column of the image in Fig. 3 that although the outline information of the tank car is more completely restored, its background is blurred, and a lot of the original texture details are smoothed out.

Finally, we compare the IR images reconstructed by the algorithm proposed along with this paper with the results of other methods, and it can be clearly seen that the network proposed in this paper reconstructs complex cluttered textures and geometrical structures better, especially the areas such as fences and branches next to the buildings (as shown in the fourth row). The cluttered areas on the grass are also reconstructed better with clearer details. Thanks to the introduction of the SCE module used in the proposed DBFE, the model presented in this paper can obtain more desirable high-resolution images, solving the blurring problem of irregular textures and geometric structures.

#### Evaluation on the second test set

In order to further validate the effectiveness of the DBFE model proposed in this paper, we do a more detailed validation on a second test set. In this set of experiments we also added the state-of-the-art infrared super-resolution algorithm LKFormer^26^ for comparison with the proposed DBFE. Table 2 shows the quantitative results of each algorithm on the IR700 dataset. From the quantitative results in Table 2, it can be seen that compared to the previous state-of-the-art methods, our DBFE shows the best results compared to the other methods.

**Table 2:** Evaluation results of each model on the second test set.

Dataset	Metrics	EDSR	SRGAN	R-ESRGAN	SwinIR	LKFormer	Ours
IR700	PSNR	31.36	30.89	31.24	32.78	32.95	33.28
	SSIM	0.8416	0.8223	0.8381	0.8405	0.8616	0.8718

**Figure 4:**
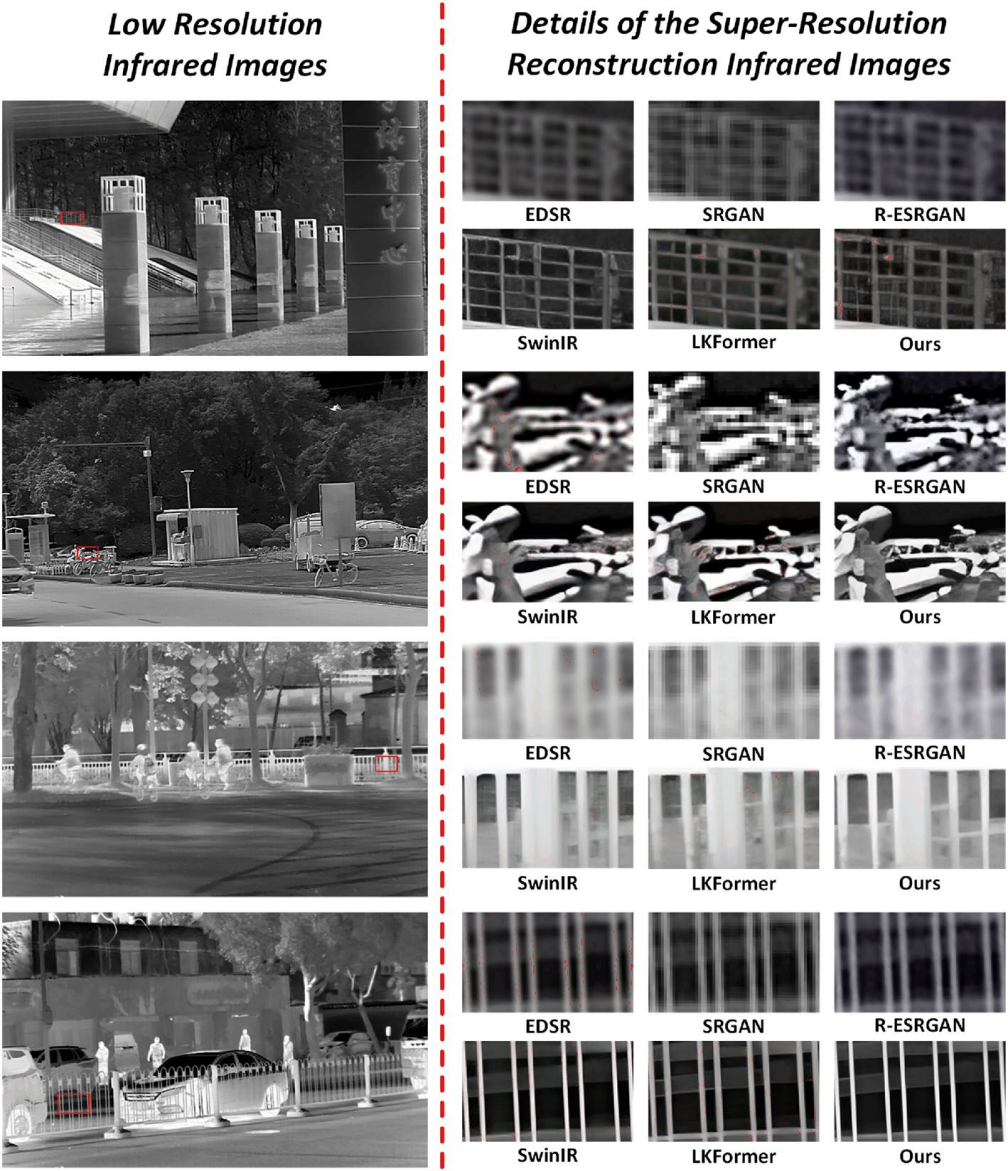
Visual comparisons of DBFE with other SRR methods on IR700 dataset.

Figure 4 shows the detail comparison of the IR images reconstructed by each model in different scenes. We can clearly see that EDSR, SRGAN and R-ESRGAN are inaccurate and not good in recovering the details of geometric structures and texture features. In contrast SwinIR and LKFormer reconstructed images with clearer details. Compared to these methods, the IR images reconstructed by DBFE proposed in this paper have the clearest geometric structure and texture features and show superior performance in different scenes.

The reason why our proposed DBFE achieves the most superior performance is that DBFE uses the STE module for the full extraction of high-frequency information and low-frequency information while increasing the local dependence and global dependence on features of different scales, and ultimately enhances the gradient magnitude of the features to enrich the feature saliency in order to reconstruct the high-frequency texture information and low-frequency structural information more accurately.

#### Component analysis

In order to further validate the effectiveness of the SCE module used in this paper, a set of ablation experiments were done. The experiments compare the reconstruction effect of the model in this paper with and without the addition of the SCE module.

As shown in Table 3, the quantitative results of the algorithm are much improved in the case of using the SCE module compared to the version without applying SCE.

**Figure 5:**
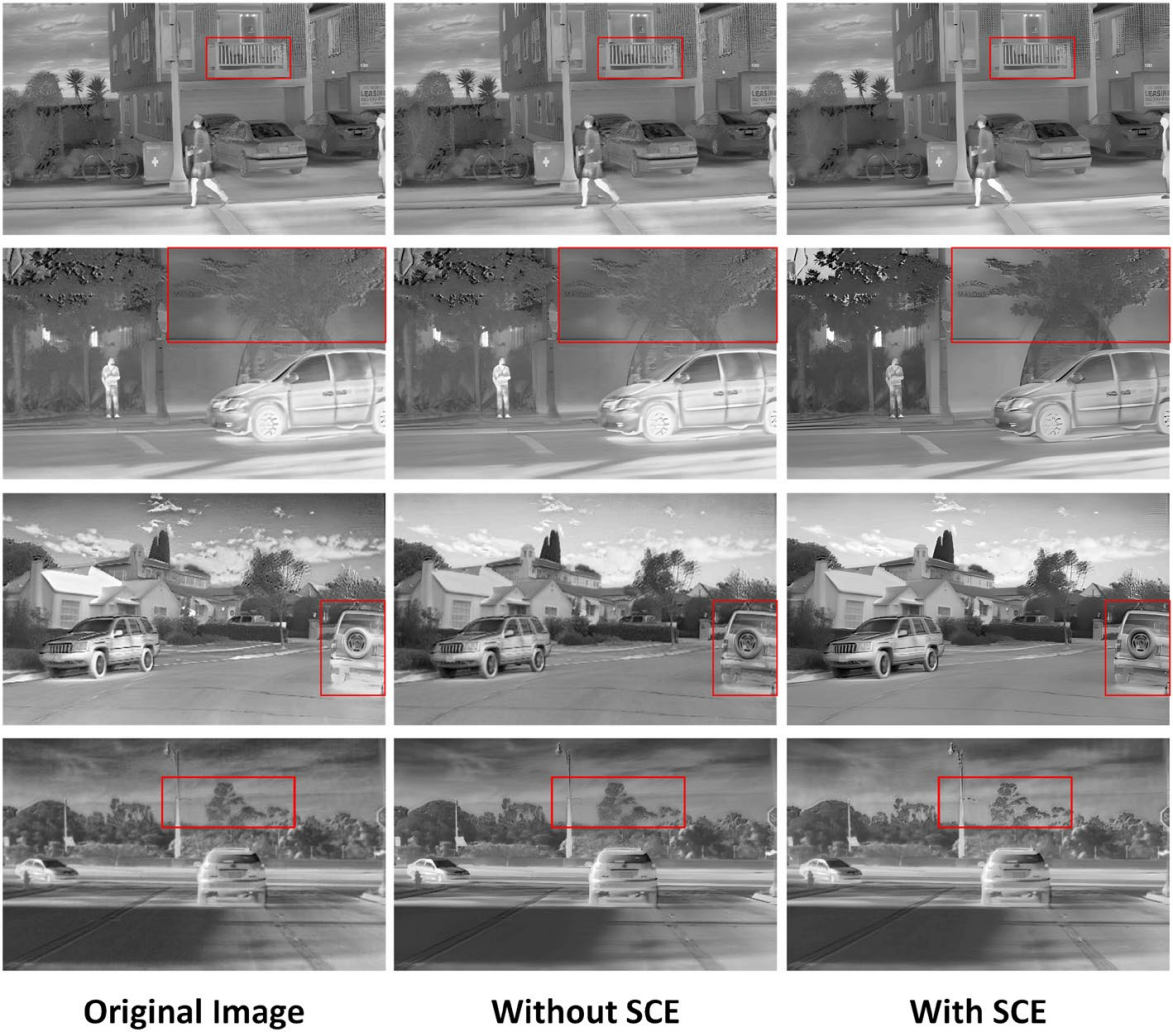
Results of qualitative analysis of ablation experiments with the SCE module.

Accordingly, the visual comparison provided in Fig. 5 also demonstrates the effectiveness of the SCE module. The original image in Fig. 5 is blurred, the contour features of the objects are not distinct and the texture details are missing. When we reconstruct the image using DBFE without the SCE module, the reconstructed image geometry is clearer. However, the reconstruction effect of the model is too smooth and a lot of texture information is lost. When we add the SCE module to the model, the reconstructed infrared image is not only geometrically clear, but also rich in texture features. In particular, comparing with the area marked by the red box in the figure, we can clearly observe that the texture features of the reconstructed image are more explicit after using the SCE module.

**Table 3:** Results of quantitative analyses of ablation experiments with the SCE module.

Networks	Dataset	PSNR	SSIM
Original image	RoadScene	22.38	0.71
	TNO	23.58	0.73
	MSRS	21.16	0.69
DBFE without SCE	RoadScene	26.80	0.82
	TNO	28.44	0.79
	MSRS	23.37	0.66
DBFE	RoadScene	28.97	0.90
	TNO	31.23	0.88
	MSRS	26.37	0.75

“DBFE without SCE” means that the SCE module has been removed from our network without any other changes. “DBFE” refers to the model proposed in this article.

## Conclusion

To address the problems of existing high-resolution reconstruction algorithms for infrared images, this paper proposes a Dual-Branch Feature Encoding Framework, which uses the Structure-Contextual Encoder module proposed in this paper to fully extract the texture features and geometric structure of different feature levels and establish the global dependency of features. global dependencies of features. We demonstrate the effectiveness of our network through an extensive evaluation of a test image set, which achieves a significant improvement in the super-resolution reconstruction of single-frame infrared images.

## Data Availability

The data generated and analysed in this study are available on request from the corresponding author on reasonable request.
